# Auricular transcutaneous vagus nerve stimulation alters directed cortical communication during intentional actions

**DOI:** 10.1016/j.isci.2025.114571

**Published:** 2025-12-29

**Authors:** Moritz Mückschel, Jasmin Mayer, Bernhard Hommel, Christian Beste

**Affiliations:** 1 Cognitive Neurophysiology, Department of Child and Adolescent Psychiatry, Faculty of Medicine, TU Dresden, Dresden, Germany; 2 School of Psychology, Shandong Normal University, Jinan, China

**Keywords:** biological sciences, neuroscience

## Abstract

Understanding how intentional behavior emerges from neural dynamics requires linking cognitive theories with neurobiology. We combined auricular transcutaneous vagus nerve stimulation (atVNS) with EEG-based directed connectivity analyses to probe action-effect integration in a canonical theta-band network comprising the anterior temporal lobe (ATL), insular cortex (IC), and inferior frontal cortex (IFC). We show that this core network supports action-effect processing, but atVNS additionally recruited posterior temporal/ventral stream regions (PTL) and altered directed information transfer in the network. While some network properties (e.g., IFC-PTL asymmetry) were involved in both action-effect perception and planning, others (e.g., IC-IFC coupling) were specific to only one of these processes, suggesting that ideomotor theory would benefit from process-specific assumptions regarding the cortical dynamics. The results can be interpreted as reflecting enhanced GABAergic transmission underlying atVNS effects, providing further neurobiological foundation for ideomotor theory on the basis of directed cortical communication and neuromodulation.

## Highlights

• AtVNS modulates the segregation of ATL, IC, and IFC theta-band networks
• Reduced directed information transfer suggests GABAergic modulation by atVNS
• Connectivity effects dissociate planning from perception, refining ideomotor theory

## Graphical abstract

**Figure:**
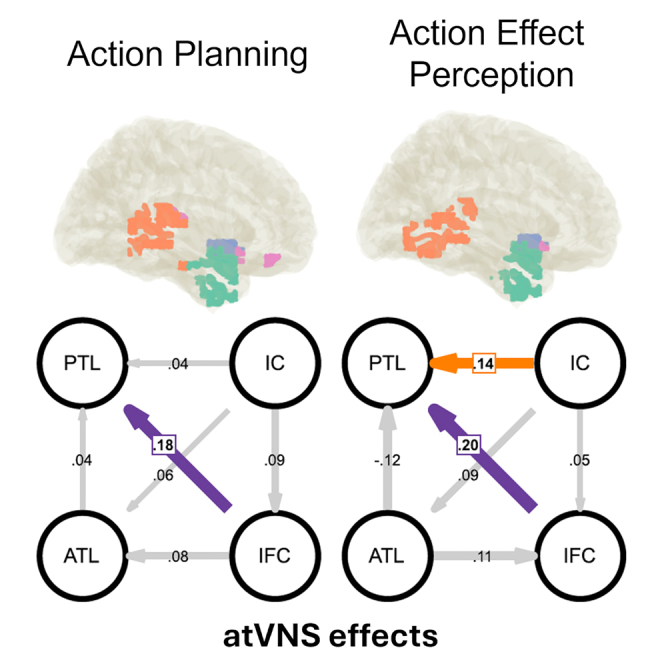

## Introduction

When humans act, they mostly do so intentionally; that is, they move to achieve the intended (sensory) effect. This implies action-effect anticipation processes, which are the core principle of ideomotor theory.^1^^,^^2^^,^^3^ According to ideomotor theorizing, agents learn to act intentionally by first acquiring associations between motor patterns and the sensory effects that these patterns produce (aka action-effect bindings, AEBs), and then reactivating the former by “thinking of” (i.e., endogenously reactivating) the latter^4^ or by encountering external stimuli that resemble these effects (Elsner & Hommel, 2001). AEBs thus reflect a fundamental brick to understanding human intentional behavior, but their neurobiological underpinnings and their mechanistic basis are not yet sufficiently understood.

Imaging studies have revealed preliminary hints of the functional neuroanatomical architecture underlying AEBs,^4^^,^^5^^,^^6^^,^^7^ and more recent studies provide evidence for specific patterns of neural oscillatory activity important for AEBs.^8^^,^^9^^,^^10^ Across studies, it has been demonstrated that there seems to be a canonical AEB network formed by the anterior temporal lobe (ATL), the inferior frontal cortex (IFC), and the insular cortex (IC) that remains stable throughout development.^8^^,^^9^ Theta band activity in this network mediates a directed transfer of information during action-effect anticipation, planning, and validation processes.^8^^,^^9^

At a neurobiological level, increasing catecholaminergic activity through the administration of a combined norepinephrine (NE)/dopamine (DA) transporter blocker (i.e., methylphenidate, MPH)^11^ changes the theta-band activity associated with directed information transfer in the AEB network^9^: During action-effect planning, increased catecholaminergic concentrations changed the network dynamics by increasing information flow toward the inferior frontal cortex (IFC). This reversed the neurophysiological pattern under placebo, where IFC mainly sent information to ATL. In the perception/validation phase, after successful production of the intended effect, the pattern reversed with increased catecholaminergic concentrations. Overall, modulating the catecholaminergic system induced a reorganization of directed connectivity in cortical theta networks serving action-effect integration. This modulation of network-level processes by catecholamines can be interpreted as reflecting an aspect of gain modulation principles, which are well known to be a function of the catecholaminergic system.^12^^,^^13^^,^^14^

Importantly, not only do catecholamines affect network-level processes, possibly through a sharpening of gain control,^13^^,^^15^^,^^16^ but the GABAergic system seems to be involved as well. The reason is that the NE and the DA system modulate GABAergic neural transmission,^17^^,^^18^^,^^19^^,^^20^ and evidence suggests that the GABAergic system itself is important for gain control.^20^^,^^21^ Therefore, it seems essential to investigate the combined effects of catecholaminergic and GABAergic neuromodulation on action-effect binding. Gain modulation has also been suggested to underlie the effect of auricular transcutaneous vagus nerve stimulation (atVNS),^22^ because atVNS likely modulates both the catecholaminergic^23^^,^^24^^,^^25^^,^^26^ and the GABAergic system.^27^^,^^28^^,^^29^^,^^30^ For a detailed discussion on the neurobiological underpinnings of atVNS, see.^22^^,^^31^^,^^32^^,^^33^ It is assumed that the concomitant modulation of the catecholaminergic and the GABAergic systems alters gain control (i.e., signal-to-noise ratio) in neural networks.^22^^,^^34^^,^^35^ Therefore, we hypothesized that atVNS affects AEB processes by modulating theta-band associated directed communication with the action-effect integration network, compared to a sham stimulation condition. This cortical action-effect integration network comprises the ATL, the IFC, and the IC,^8^^,^^10^ and atVNS alters the direction of communication within this network. Changes in the directed network communication can be expressed in changes in the strength of information transfer from brain region “A” to brain region “B” and from brain region “B“ to “A.” Thus, not only can the strength of information transfer between regions change because of atVNS, but also the symmetry/asymmetry of information transfer between regions. We examined both aspects in the present study.

**Figure 1:**
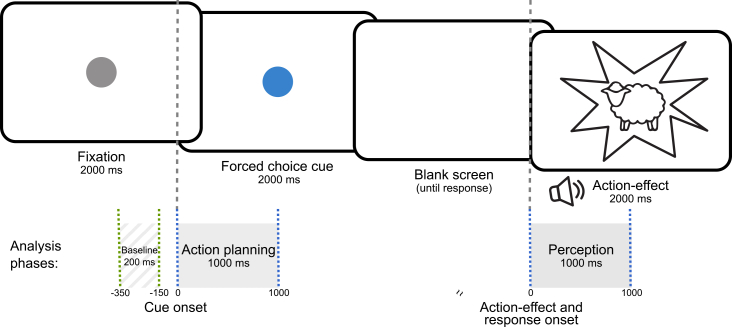
Experimental setup Schematic depiction of the action-effect binding experiment. Green dashed lines indicate the baseline interval relative to the onset of the cue stimulus. Blue dashed lines show the time interval of the two analysis time windows: the action planning phase relative to the onset of the cue and the perception phase relative to the action-effect onset. The scaling of the time axis is not proportional.

More specifically, we employed EEG frequency tagging as an experimental approach for action-effect integration.^8^^,^^9^^,^^36^ In this procedure, a motor response induces a flickering of visual action effects (key-press-produced pictures) at a pre-defined frequency (see Figure 1 for the task). Following the acquisition of an action, increased brain activity is observed at the frequency corresponding to the previously entrained action effect, indicating anticipatory activation of the action-effect before the action is performed.^8^^,^^9^^,^^36^ Thus, action effects are not measured behaviorally but are objectified using basic neurophysiological principles. We used theta band activity (4–7 Hz) and included an 8 Hz flickering control condition (for details, see the method details section). This procedure is identical to the one used by Mayer et al.^8^ To investigate how atVNS affects directed transfer of information during action-effect integration in the ATL-IFC-IC network, we used nonlinear causal relationship estimation by artificial neural network (nCREANN).^37^ We distinguished between two theoretically relevant time periods, the planning period, during which agents were assumed to reactivate AEBs in order to anticipate the intended action effects and prepare the corresponding motor patterns, and the perception period, during which they were processing the self-produced action effect.

## Results

### Subjective reports on the auricular transcutaneous vagus nerve stimulation procedure

The atVNS side effects as reported by the participants were compared between the sham and atVNS conditions using Chi-Square tests. Since the self-reports were incomplete for three participants, the tests were conducted for a subsample of *n* = 47 participants. The mean subjective ratings for the side effects are given in Table 1.

**Table 1:** Results of atVNS side effects scale

Symptom	n	atVNS	Sham	p	p (FDR)
Headache	47	1.19 ± 0.06	1.3 ± 0.07	0.332	0.498
Neck pain	47	1.28 ± 0.07	1.4 ± 0.1	0.241	0.45
Nausea	47	1.02 ± 0.02	1.11 ± 0.05	0.25	0.45
Muscle Contraction in the face or neck	47	1.21 ± 0.07	1.32 ± 0.1	0.227	0.45
Stinging sensation	47	1.72 ± 0.13	1.49 ± 0.1	0.051	0.229
Burning sensation	47	1.38 ± 0.09	1.17 ± 0.06	0.046	0.229
General Discomfort	47	1.43 ± 0.09	1.36 ± 0.08	0.563	0.658
Other sensations and/or aversive effects	47	1.28 ± 0.1	1.26 ± 0.11	0.973	0.973
Total score	47	10.51 ± 0.38	10.4 ± 0.37	0.585	0.658

atVNS side effects were assessed via a self-report questionnaire in the atVNS as well as the sham condition. Participants were asked to report their symptoms on a 5-item Likert-scale, where 1 is “not at all,” and 5 is “Very strong.” For each symptom as well as the total (summed up) score, the mean and standard error of the mean are given. Additionally, the *p* value and the FDR-corrected *p* value based on Chi-Square tests are given.

Statistical analysis revealed that significant stimulation effects were only observed for the symptom “burning sensation,” but this effect did not survive FDR correction,^38^ *p* = 0.046; p after FDR = 0.229. Additionally, participants were asked to guess the time point of active stimulation. To assess whether connectivity (see sections later in discussion) differed between participants who correctly identified the stimulation time point and those who did not, two-sample t-tests were conducted on linear and non-linear connectivities across all conditions and analysis phases (see Table S1). Of 96 FDR-corrected tests, only one showed significant group differences (*p* = 0.002). All other tests were not significant. Importantly, this suggests that although sham blinding was not fully successful according to the active stimulation estimation, any resulting effects are minimal.

### Behavioral results

As shown by paired-sample t-tests, there is no significant effect of stimulation on the reaction times (t(49) = −1.58; *p* = 0.12; d = 0.22; atVNS: 304.12 ± 6.72; sham: 310.17 ± 7.58). Additionally, there is no significant effect of stimulation on accuracy (t(49) = 1.54; *p* = 0.13; d = 0.22; atVNS: 0.97 ± 0; sham: 0.97 ± 0). The primary measures of action effect integration processes were the frequency tagging responses, rather than behavioral performance (see also our previous work by Mayer et al.^8^^,^^9^^,^^10^ The absence of stimulation effects on RTs or accuracy, despite clear changes in network dynamics (see later in discussion), reveals that neuromodulatory influences can reconfigure neural computations without necessarily impacting overt performance. The current paradigm yields very high accuracy and relatively fast responses, likely leaving limited room for modulation at the behavioral level. In such cases, neural measures can be more sensitive than aggregate behavioral readouts, capturing shifts in how the system solves the task.

### Neurophysiological results

**Figure 2:**
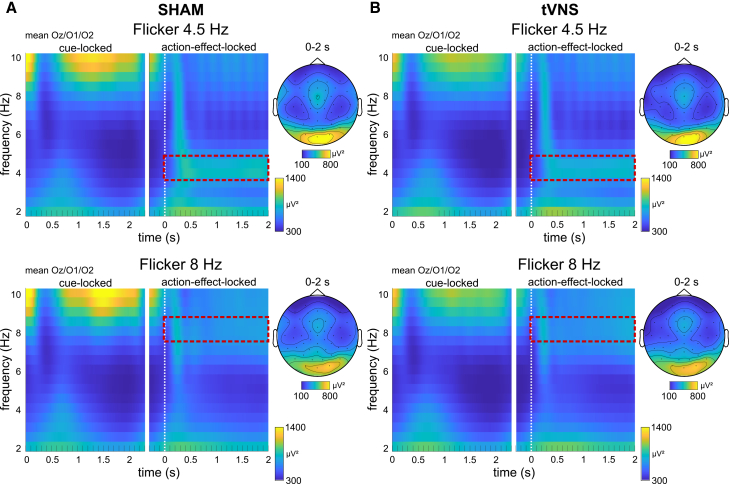
Time-frequency results The mean time-frequency power of the action-effect flicker frequency at 4.5 Hz and 8 Hz for the sham condition (left, A) and the atVNS active stimulation condition (right, B). The averaged power values of electrodes Oz, O1, and O2 are shown, in a time window of 0–2.3 s relative to the onset of the cue-stimulus and −0.3 to 2 s relative to the action-effect onset (white dotted line). The topography plots depict the power distribution at the respective action-effect frequency averaged from 0 to 2 s relative to action-effect onset (red dashed box). Data are represented as the mean.

Mean power modulations at pooled channels Oz, O1, and O2, for sham and tVNS condition, and at action effect flicker frequencies of 4.5 Hz and 8 Hz are depicted in Figure 2.

**Figure 3:**
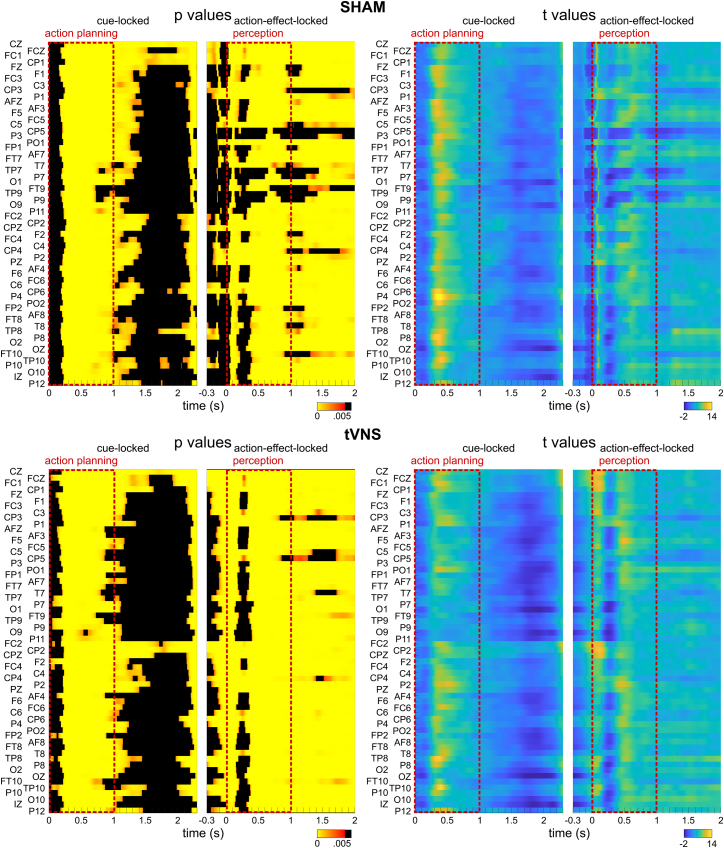
Relative power changes Results of t-tests comparing relative power changes at 4.5 Hz and 8 Hz (i.e., measured frequencies) at a flicker frequency of 4.5 Hz, with the results for the sham condition on top and the atVNS condition below. For cue-locked data, time-points between 0 s and 2.3 s were compared, and for action-effect-locked time points between −0.3 s and 2 s. P-values (left) and t-values (right) are depicted by colors, non-significant *p*-values after FDR correction are indicated by black areas. The data were segmented into two different analysis phases: 1. “action planning” phase relative to the cue stimulus onset. 2. “perception phase” relative to the action-effect onset.

Following the analysis approach of Mayer et al.^8^^,^^9^ all consecutive analysis steps focused on the 4.5 Hz flicker frequency data. Relative power changes were compared between 4.5 Hz and 8 Hz by means of paired-sample t-tests for each data point and each electrode (see Figure 3). For data locked on the cue onset the time window was 0–2300 ms. For data locked to the onset of the action effect a time window of −300 to 2000 ms was selected. FDR correction^39^ was applied to account for multiple testing. Starting at about 200 ms relative to the cue onset, significant power changes are evident in the sham condition (max *p* = 0.002; min t = 3.(19) as well as in the atVNS condition (max *p* = 0.002; min t = 3.(21) at all channels. These significant power changes are stable until about 750–1500 ms, depending on the channel. The time window until 2000 ms shows almost no significant differences. Based on action-effect onset locking, significant differences can be observed starting around action effect onset until 2000 ms in the sham condition (max *p* = 0.003; min t = 3.07) as well as in the atVNS condition (max *p* = 0.004; min t = 3.02), with a small gap between about 200 and 400 ms. For further analysis, the time window of 0–1000 ms relative to the cue onset (“action planning phase”) as well as 0 to 1000 ms relative to the action-effect onset (“action phase”) was selected.

### Source localization and regions of interest selection

**Figure 4:**
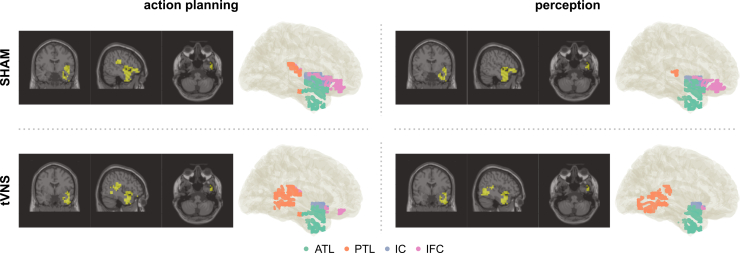
DICS source localization DICS beamformer results at 4.5 Hz and action-effect of 4.5 Hz for the action planning and perception phase in the sham condition (top) and the atVNS condition (bottom). Only the top 3% of voxels by source activity are shown in the orthographic plots, revealing right-hemisphere sources in temporal, inferior frontal, and insular areas. Additionally, the four defined regions of interest are depicted in different colors in a glass brain: anterior temporal lobe (ATL), posterior temporal lobe (PTL), inferior frontal cortex (IFC), and insular cortex (IC).

A DICS beamformer was applied to localize cortical sources of theta-band activity at 4.5 Hz for each analysis phase, as described in the STAR Methods section. For each phase, the top 3% of voxels contributing most strongly to theta activity were selected and assigned anatomical labels. This choice reflects a trade-off between focusing the directed connectivity analysis on the most prominent cortical activity, while still allowing degrees of freedom that multiple cortical activity clusters emerge, possibly reflecting networked activity. Voxels located outside the brain, within white matter, or in the cerebellum were excluded. To further refine the selection, a DBSCAN clustering algorithm was used to group voxels into clusters, as shown in Figure 4. The clustered voxels were then manually segmented into four functional regions of interest (ROI). Isolated voxels that did not belong to any cluster, as well as voxels within subcortical structures such as the basal ganglia, were discarded due to the limited accuracy of EEG source estimation in small or deep brain regions. Across phases, this procedure consistently yielded three right-hemisphere ROIs: the anterior temporal lobe (ATL), the posterior temporal lobe (PTL), the inferior frontal cortex (IFC), and the insular cortex (IC). ATL and PTL voxels were separated based on the median MNI y-coordinate of Heschl’s gyrus. Labeling of voxels was done using the Automated Anatomical Labeling (AAL) atlas, version 3.^40^ The ATL and PTL encompassed right-hemisphere voxel with labels corresponding to inferior temporal gyrus, temporal pole (middle temporal gyrus), superior temporal gyrus, middle temporal gyrus, temporal pole (superior temporal gyrus) and parahippocampal gyrus. For the IC, all voxels corresponding to the insula were included. The IFC comprised voxels in opercular and triangular part of inferior frontal gyrus, pars orbitalis of the inferior frontal gyrus, and the rolandic operculum.

Because of the limited spatial resolution of EEG and the relatively deep and gyral anatomy of the insular cortex, the separation of IC and adjacent frontal opercular sources is necessarily approximate. The DICS beamformer and ROI definitions, therefore, provide functionally meaningful but not anatomically perfect isolation of IC activity.

### Directed connectivity analyses

Directed connectivity, both linear and non-linear, between the reconstructed sources was assessed using nCREANN analysis.^8^^,^^37^^,^^41^^,^^42^ Model performance was robust, as reflected in low mean squared errors (MSEs) for both training (atVNS: 0.03 ± 0.02 SD; sham: 0.04 ± 0.02) and testing (atVNS: 0.04 ± 0.02; sham: 0.04 ± 0.03) datasets. Model fit was high, with mean R^2^ values of 0.97 ± 0.06 for training and 0.97 ± 0.06 for the testing of atVNS data, as well as 0.96 ± 0.06 for training and 0.96 ± 0.06 for the testing of sham data. Training and testing R^2^ values were strongly positively correlated (r > 0.85, *p* < 0.001). To ensure that only genuine directed interactions were analyzed, all connectivity values were first tested against surrogate-data distributions (see STAR Methods section for details), and only connections exceeding the surrogate null were entered into group-level statistical comparisons. Subsequent stimulation contrasts were corrected using FDR correction.

**Figure 5:**
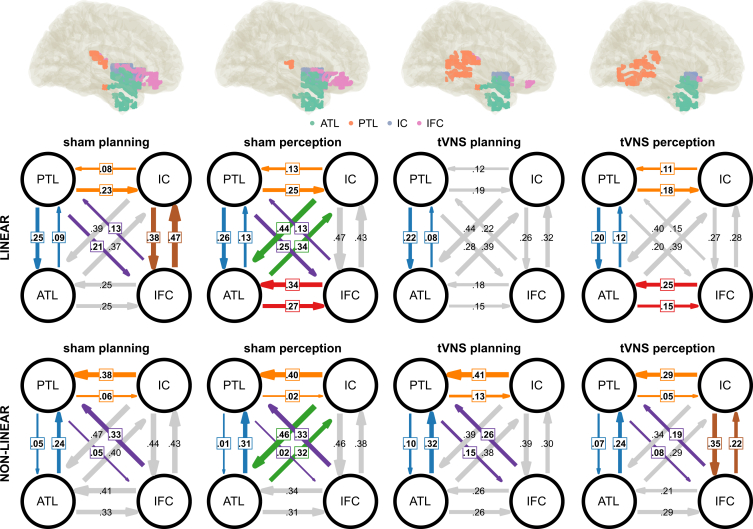
nCREANN connectivities and bilateral asymmetries Linear and non-linear effective connectivity patterns as revealed by nCREANN. For each phase, linear and non-linear connectivity patterns between established ROIs (ATL: anterior temporal lobe; PTL: posterior temporal lobe; IC: insular cortex; IFC: inferior frontal cortex) are depicted (i.e., mean connectivity strength). The direction of input flows between two ROIs is shown by arrows. The connectivity strengths are depicted by the thickness of the arrows. Significant differences between bidirectional connectivities after FDR correction (α = 0.05) are indicated by colored arrows and text boxes. Non-significant connection differences are indicated by gray arrows. Data are represented as mean.

All linear connectivities ranged from 0.08 to 0.48 (mean: 0.25 ± 0.01). In a first step, the bilateral connectivities (i.e., ROI A to B versus ROI B to A) were compared using paired-sample and Bayesian t-tests. Detailed results are given in Table 2 for linear connectivities and Table 3 for non-linear connectivities. Additionally, the linear and non-linear connectivities for action planning and perception phase as well as sham and tVNS condition are depicted in Figure 5. The strength of the connectivities is depicted by the thickness of the respective arrows. Significant bilateral connectivity differences are indicated by colored arrows as well as a colored text box, whereas non-significant connectivity differences are depicted with gray arrows. FDR correction has been applied to account for multiple testing.

**Table 2:** Linear bidirectional connectivities

Phase	Connection	ROI a → b	ROI b → a	t	d	p	p (FDR)	BF10	BF evidence
**SHAM**									

planning	ATL <> PTL	0.09 ± 0.02	0.25 ± 0.03	−5.68	0.8	0	0	21876.72	extreme H1
planning	ATL <> IC	0.37 ± 0.05	0.39 ± 0.05	−0.63	0.09	0.534	0.611	0.19	moderate H0
planning	ATL <> IFC	0.25 ± 0.04	0.25 ± 0.04	−0.06	0.01	0.952	0.952	0.15	moderate H0
planning	PTL <> IC	0.23 ± 0.03	0.08 ± 0.02	4.98	0.7	0	0	2257.75	extreme H1
planning	PTL <> IFC	0.21 ± 0.03	0.13 ± 0.02	2.56	0.36	0.014	0.032	2.9	anecdotal H1
planning	IC <> IFC	0.38 ± 0.05	0.47 ± 0.05	−2.42	0.34	0.019	0.039	2.13	anecdotal H1
perception	ATL <> PTL	0.13 ± 0.03	0.26 ± 0.02	−3.69	0.52	0.001	0.003	47.47	very strong H1
perception	ATL <> IC	0.34 ± 0.04	0.44 ± 0.04	−2.42	0.34	0.019	0.039	2.13	anecdotal H1
perception	ATL <> IFC	0.27 ± 0.04	0.34 ± 0.04	−2.31	0.33	0.025	0.047	1.71	anecdotal H1
perception	PTL <> IC	0.25 ± 0.02	0.13 ± 0.02	3.56	0.5	0.001	0.003	33.67	very strong H1
perception	PTL <> IFC	0.25 ± 0.02	0.13 ± 0.03	3.42	0.48	0.001	0.004	22.72	strong H1
perception	IC <> IFC	0.47 ± 0.04	0.43 ± 0.04	1.28	0.18	0.207	0.276	0.33	moderate H0

**atVNS**									

planning	ATL <> PTL	0.08 ± 0.02	0.22 ± 0.03	−3.74	0.53	0	0.003	55.01	very strong H1
planning	ATL <> IC	0.39 ± 0.05	0.44 ± 0.05	−1.03	0.15	0.307	0.388	0.25	moderate H0
planning	ATL <> IFC	0.15 ± 0.03	0.18 ± 0.03	−0.98	0.14	0.334	0.401	0.24	moderate H0
planning	PTL <> IC	0.19 ± 0.03	0.12 ± 0.02	2.09	0.3	0.042	0.067	1.12	anecdotal H1
planning	PTL <> IFC	0.28 ± 0.04	0.22 ± 0.04	1.59	0.22	0.119	0.168	0.5	anecdotal H0
planning	IC <> IFC	0.26 ± 0.03	0.32 ± 0.04	−1.64	0.23	0.107	0.161	0.54	anecdotal H0
perception	ATL <> PTL	0.12 ± 0.02	0.20 ± 0.03	−3.05	0.43	0.004	0.011	9.03	moderate H1
perception	ATL <> IC	0.39 ± 0.05	0.40 ± 0.05	−0.28	0.04	0.781	0.815	0.16	moderate H0
perception	ATL <> IFC	0.15 ± 0.03	0.25 ± 0.03	−2.82	0.4	0.007	0.018	5.16	moderate H1
perception	PTL <> IC	0.18 ± 0.02	0.11 ± 0.02	3.55	0.5	0.001	0.003	32.61	very strong H1
perception	PTL <> IFC	0.20 ± 0.03	0.15 ± 0.03	2.2	0.31	0.033	0.056	1.38	anecdotal H1
perception	IC <> IFC	0.27 ± 0.03	0.28 ± 0.04	−0.38	0.05	0.706	0.77	0.16	moderate H0

Linear connectivities and results of frequentist and Bayesian *t* test comparing bidirectional connectivities for action planning and perception phase. Mean and SEM are given for each connection beside t-value (t), Cohen’s d (d), *p*-value (p), and FDR corrected *p*-value (p (FDR)). The Bayes factor strength of evidence label is given as evidence toward H1 if BF10 > 1 and otherwise as evidence toward H0 based on Lee and Wagenmakers.^43^

**Table 3:** Non-linear bidirectional connectivities

Phase	Connection	ROI a → b	ROI b → a	t	d	p	p (FDR)	BF10	BF evidence
**SHAM**									

planning	ATL <> PTL	0.24 ± 0.04	0.05 ± 0.01	4.41	0.62	0	0	387.8	extreme H1
planning	ATL <> IC	0.40 ± 0.05	0.47 ± 0.04	−1.16	0.16	0.251	0.317	0.29	moderate H0
planning	ATL <> IFC	0.33 ± 0.04	0.41 ± 0.04	−1.72	0.24	0.091	0.121	0.61	anecdotal H0
planning	PTL <> IC	0.06 ± 0.01	0.38 ± 0.04	−7.92	1.12	0	0	4.22∗10^−7^	extreme H1
planning	PTL <> IFC	0.05 ± 0.01	0.33 ± 0.04	−7.2	1.02	0	0	3.64∗10^−6^	extreme H1
planning	IC <> IFC	0.44 ± 0.04	0.43 ± 0.04	0.01	0	0.995	0.995	0.15	moderate H0
perception	ATL <> PTL	0.31 ± 0.05	0.01 ± 0.01	6.22	0.88	0	0	1.34∗10^−5^	extreme H1
perception	ATL <> IC	0.32 ± 0.05	0.46 ± 0.04	−2.55	0.36	0.014	0.024	2.85	anecdotal H1
perception	ATL <> IFC	0.31 ± 0.04	0.34 ± 0.04	−0.76	0.11	0.452	0.517	0.2	moderate H0
perception	PTL <> IC	0.02 ± 0.01	0.40 ± 0.04	−9.42	1.33	0	0	5.92∗10^−9^	extreme H1
perception	PTL <> IFC	0.02 ± 0.01	0.33 ± 0.04	−7.12	1.01	0	0	2.82∗10^−6^	extreme H1
perception	IC <> IFC	0.46 ± 0.04	0.38 ± 0.04	1.9	0.27	0.064	0.096	0.8	anecdotal H0

**atVNS**									

planning	ATL <> PTL	0.32 ± 0.05	0.10 ± 0.02	5.38	0.76	0	0	8185.98	extreme H1
planning	ATL <> IC	0.38 ± 0.05	0.39 ± 0.04	−0.24	0.03	0.814	0.888	0.16	moderate H0
planning	ATL <> IFC	0.26 ± 0.04	0.26 ± 0.03	0.03	0	0.977	0.995	0.15	moderate H0
planning	PTL <> IC	0.13 ± 0.02	0.41 ± 0.04	−7.11	1.01	0	0	2.69∗10^−6^	extreme H1
planning	PTL <> IFC	0.15 ± 0.03	0.26 ± 0.03	−3.33	0.47	0.002	0.004	17.94	strong H1
planning	IC <> IFC	0.39 ± 0.04	0.30 ± 0.03	1.75	0.25	0.087	0.121	0.63	anecdotal H0
perception	ATL <> PTL	0.24 ± 0.04	0.07 ± 0.03	3.78	0.53	0	0.001	61.72	very strong H1
perception	ATL <> IC	0.29 ± 0.05	0.34 ± 0.04	−1.05	0.15	0.299	0.359	0.26	moderate H0
perception	ATL <> IFC	0.29 ± 0.05	0.21 ± 0.02	1.99	0.28	0.052	0.084	0.94	anecdotal H0
perception	PTL <> IC	0.05 ± 0.02	0.29 ± 0.04	−6.56	0.93	0	0	4.19∗10^−5^	extreme H1
perception	PTL <> IFC	0.08 ± 0.03	0.19 ± 0.03	−3.09	0.44	0.003	0.007	9.89	moderate H1
perception	IC <> IFC	0.35 ± 0.04	0.22 ± 0.03	3	0.42	0.004	0.008	7.87	moderate H1

Non-linear connectivities and results of frequentist and Bayesian *t* test comparing bidirectional connectivities for action planning and perception phase. Mean and SEM are given for each connection beside t-value (t), Cohen’s d (d), *p*-value (p), and FDR corrected *p*-value (p (FDR)). The Bayes factor strength of evidence label is given as evidence toward H1 if BF10 > 1 and otherwise as evidence toward H0 based on Lee and Wagenmakers.^43^

Across analyses, we identified a stable theta-band core network comprising the anterior temporal lobe (ATL), insular cortex (IC), and inferior frontal cortex (IFC) supporting action-effect integration. Active atVNS modulated this network in three characteristic ways: (i) it recruited an additional posterior temporal/ventral-stream node (PTL), (ii) it selectively reduced directed information transfer between IC→IFC and IFC→ATL during perception, and (iii) it altered bilateral asymmetries in PTL-IFC connectivity across both planning and perception phases.

### Linear directed connectivities

During the sham condition in the action planning phase, the linear connectivity of PTL to ATL (0.25 ± 0.03) was significantly larger (*p* < 0.001) than the connectivity of ATL to PTL (0.09 ± 0.02). Likewise, PTL to IC connectivity (0.23 ± 0.03) was significantly larger (*p* < 0.001) than IC to PTL (0.08 ± 0.02). PTL to IFC connectivity (0.21 ± 0.03) was significantly larger (*p* = 0.032) than IFC to PTL (0.13 ± 0.02). Additionally, IFC to IC connectivity (0.47 ± 0.05) was significantly larger (*p* = 0.039) than IC to IFC (0.38 ± 0.05). All other connectivities during the sham condition action planning phase did not differ significantly.

During the perception phase, PTL to ATL linear connectivity (0.26 ± 0.02) was significantly larger (*p* = 0.003) than ATL to PTL (0.13 ± 0.03). Similarly, IC to ATL connectivity (0.44 ± 0.04) was significantly larger (*p* = 0.039) than ATL to IC (0.34 ± 0.04), and IFC to ATL connectivity (0.34 ± 0.04) was significantly larger (*p* = 0.047) than ATL to IFC (0.27 ± 0.04). PTL to IC connectivity (0.25 ± 0.02) was significantly larger (*p* = 0.003) than IC to PTL (0.13 ± 0.02). PTL to IFC connectivity (0.25 ± 0.02) was significantly larger (*p* = 0.004) than IFC to PTL (0.13 ± 0.03). The connectivity between IC and IFC did not differ significantly.

During the atVNS condition in the action planning phase, ATL to PTL linear connectivity (0.22 ± 0.03) was significantly larger (*p* = 0.003) than PTL to ATL (0.08 ± 0.02). All other connectivities did not differ significantly. In the perception phase, ATL to PTL connectivity (0.20 ± 0.03) was significantly larger (*p* = 0.011) than PTL to ATL (0.12 ± 0.02). IFC to ATL connectivity (0.25 ± 0.03) was significantly larger (*p* = 0.018) than ATL to IFC (0.15 ± 0.03), and PTL to IC connectivity (0.18 ± 0.02) was significantly larger (*p* = 0.003) than IC to PTL (0.11 ± 0.02). The connectivity between PTL and IFC as well as IC and IFC did not differ significantly. For linear connectivities, detailed results are given in Table 2.

### Non-linear directed connectivities

The non-linear connectivities ranged from 0.01 to 0.47 (mean: 0.27 ± 0.02). During the sham condition in the action planning phase, ATL to PTL connectivity (0.24 ± 0.04) was significantly larger (*p* < 0.001) than PTL to ATL (0.05 ± 0.01). IC to PTL connectivity (0.38 ± 0.04) was significantly larger (*p* < 0.001) than PTL to IC (0.06 ± 0.01), and IFC to PTL connectivity (0.33 ± 0.04) was significantly larger (*p* < 0.001) than PTL to IFC (0.05 ± 0.01). All other connectivities during sham action planning did not differ significantly. In the sham perception phase, ATL to PTL connectivity (0.31 ± 0.05) was significantly larger (*p* < 0.001) than PTL to ATL (0.01 ± 0.01). IC to ATL connectivity (0.46 ± 0.04) was significantly larger (*p* = 0.024) than ATL to IC (0.32 ± 0.05). In addition, IC to PTL connectivity (0.40 ± 0.04) was significantly larger (*p* < 0.001) than PTL to IC (0.02 ± 0.01), and IFC to PTL connectivity (0.33 ± 0.04) was significantly larger (*p* < 0.001) than PTL to IFC (0.02 ± 0.01). All other connectivities during sham perception did not differ significantly.

During the tVNS condition in the action planning phase, ATL to PTL connectivity (0.32 ± 0.05) was significantly larger (*p* < 0.001) than PTL to ATL (0.10 ± 0.02). IC to PTL connectivity (0.41 ± 0.04) was significantly larger (*p* < 0.001) than PTL to IC (0.13 ± 0.02), and IFC to PTL connectivity (0.26 ± 0.03) was significantly larger (*p* = 0.004) than PTL to IFC (0.15 ± 0.03). All other connectivities during atVNS action planning did not differ significantly. In the tVNS perception phase, ATL to PTL connectivity (0.24 ± 0.04) was significantly larger (*p* = 0.001) than PTL to ATL (0.07 ± 0.03). IC to PTL connectivity (0.29 ± 0.04) was significantly larger (*p* < 0.001) than PTL to IC (0.05 ± 0.02), IFC to PTL connectivity (0.19 ± 0.03) was significantly larger (*p* = 0.007) than PTL to IFC (0.08 ± 0.03), and IFC to IC connectivity (0.35 ± 0.04) was significantly larger (*p* = 0.008) than IC to IFC (0.22 ± 0.03). All other connectivities during tVNS perception did not differ significantly. For linear connectivities, detailed results are given in Table 3.

### Single directed connectivities stimulation effects

**Figure 6:**
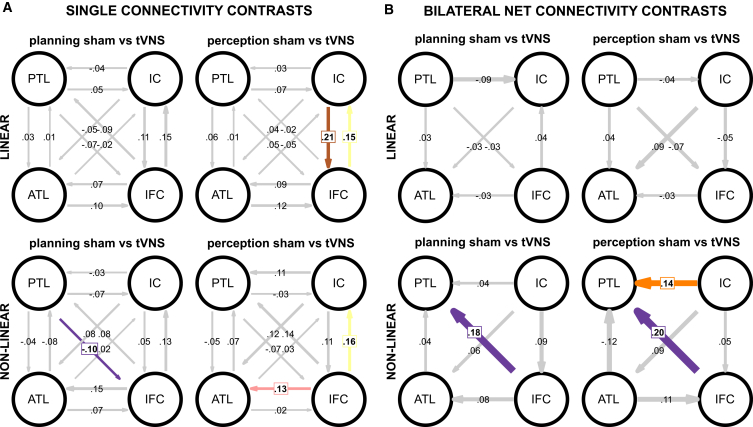
nCREANN connectivities stimulation effects Effect of stimulation (sham versus atVNS condition) on linear and non-linear connectivities between the four established ROIs (ATL: anterior temporal lobe; PTL: posterior temporal lobe; IC: insular cortex; IFC: inferior frontal cortex). (A) Single connectivity contrast. The arrow thickness and direction indicate the mean stimulation effect on input flow. Significant differences between sham and atVNS conditions after FDR correction (α = 0.05) are indicated by colored arrows and text boxes. Non-significant differences are indicated by gray arrows. (B) Bilateral net connectivity contrast. The arrow thickness indicates the mean stimulation effect on net differences, i.e., the bilateral connectivity differences compared between atVNS and the sham condition. Significant net differences after FDR correction (α = 0.05) are indicated by colored arrows and text boxes. Non-significant differences are indicated by gray arrows. Data are represented as the mean.

Next, we compared each single connectivity value between the sham and atVNS conditions using paired-sample t-tests. Detailed results are given in Table 4 for linear connectivities and Table 5 for non-linear connectivities. Additionally, the sham versus atVNS condition contrasts are depicted in Figure 6A. The strength of the connectivities is depicted by the thickness of the respective arrows. Significant stimulation condition differences are indicated by colored arrows as well as a colored text-box, whereas non-significant connectivity differences are depicted with gray arrows. FDR correction has been applied to account for multiple testing.

**Table 4:** Contrasts of linear connectivities

Connectivity	Difference	t	d	p	p (FDR)	BF10	BF evidence
**sham planning vs. atVNS planning**							

ATL > PTL	0.01 ± 0.02	0.39	0.06	0.695	0.926	0.17	moderate H0
ATL > IC	−0.02 ± 0.06	−0.41	0.06	0.681	0.926	0.17	moderate H0
ATL > IFC	0.10 ± 0.04	2.49	0.35	0.016	0.085	2.51	anecdotal H1
PTL > ATL	0.03 ± 0.04	0.99	0.14	0.325	0.522	0.25	moderate H0
PTL > IC	0.05 ± 0.04	1.35	0.19	0.183	0.367	0.36	anecdotal H0
PTL > IFC	−0.07 ± 0.04	−1.64	0.23	0.108	0.247	0.53	anecdotal H0
IC > ATL	−0.05 ± 0.05	−0.99	0.14	0.327	0.522	0.24	moderate H0
IC > PTL	−0.04 ± 0.02	−1.7	0.24	0.096	0.247	0.58	anecdotal H0
IC > IFC	0.11 ± 0.05	2.11	0.3	0.04	0.161	1.17	anecdotal H1
IFC > ATL	0.07 ± 0.04	1.64	0.23	0.108	0.247	0.53	anecdotal H0
IFC > PTL	−0.09 ± 0.03	−2.71	0.38	0.009	0.085	4.05	moderate H1
IFC > IC	0.15 ± 0.06	2.51	0.36	0.015	0.085	2.62	anecdotal H1

**sham perception vs. atVNS perception**							

ATL > PTL	0.01 ± 0.04	0.41	0.06	0.681	0.909	0.17	moderate H0
ATL > IC	−0.05 ± 0.06	−0.77	0.11	0.445	0.711	0.2	moderate H0
ATL > IFC	0.12 ± 0.05	2.48	0.35	0.017	0.088	2.44	anecdotal H1
PTL > ATL	0.06 ± 0.04	1.54	0.22	0.13	0.346	0.46	anecdotal H0
PTL > IC	0.07 ± 0.03	2.26	0.32	0.028	0.112	1.57	anecdotal H1
PTL > IFC	0.05 ± 0.03	1.39	0.2	0.171	0.392	0.38	anecdotal H0
IC > ATL	0.04 ± 0.05	0.81	0.12	0.419	0.711	0.21	moderate H0
IC > PTL	0.03 ± 0.03	0.91	0.13	0.365	0.711	0.23	moderate H0
IC > IFC	0.21 ± 0.05	3.97	0.56	0	0.004	104.58	extreme H1
IFC > ATL	0.09 ± 0.05	1.86	0.26	0.068	0.218	0.76	anecdotal H0
IFC > PTL	−0.02 ± 0.04	−0.42	0.06	0.68	0.909	0.17	moderate H0
IFC > IC	0.15 ± 0.05	2.94	0.42	0.005	0.04	6.85	moderate H1

Comparison of single linear connectivity values between atVNS and sham condition for action planning and perception phase, using frequentist and Bayesian *t* test. Mean difference and SEM are given for each contrast beside t-value (t), Cohen’s d (d), *p*-value (p), and FDR corrected *p*-value (p (FDR)). The Bayes factor strength of evidence label is given as evidence toward H1 if BF10 > 1 and otherwise as evidence toward H0 based on Lee and Wagenmakers.^43^

**Table 5:** Contrasts of non-linear connectivities

Connectivity	Difference	t	d	p	p (FDR)	BF10	BF evidence
**sham planning vs. atVNS planning**							

ATL > PTL	−0.08 ± 0.06	−1.36	0.19	0.179	0.445	0.37	anecdotal H0
ATL > IC	0.02 ± 0.07	0.27	0.04	0.788	1	0.16	moderate H0
ATL > IFC	0.07 ± 0.07	0.97	0.14	0.337	0.6	0.24	moderate H0
PTL > ATL	−0.04 ± 0.02	−1.82	0.26	0.075	0.239	0.71	anecdotal H0
PTL > IC	−0.07 ± 0.03	−2.36	0.33	0.022	0.118	1.91	anecdotal H1
PTL > IFC	−0.10 ± 0.03	−3.26	0.46	0.002	0.032	15.2	strong H1
IC > ATL	0.08 ± 0.06	1.24	0.17	0.222	0.445	0.31	moderate H0
IC > PTL	−0.03 ± 0.06	−0.47	0.07	0.637	0.927	0.17	moderate H0
IC > IFC	0.05 ± 0.06	0.8	0.11	0.426	0.681	0.21	moderate H0
IFC > ATL	0.15 ± 0.05	2.74	0.39	0.009	0.068	4.3	moderate H1
IFC > PTL	0.08 ± 0.06	1.31	0.19	0.195	0.445	0.35	anecdotal H0
IFC > IC	0.13 ± 0.06	2.22	0.31	0.031	0.124	1.45	anecdotal H1

**sham perception vs. atVNS perception**							

ATL > PTL	0.07 ± 0.07	1.00	0.14	0.321	0.514	0.25	moderate H0
ATL > IC	0.03 ± 0.07	0.49	0.07	0.629	0.914	0.17	moderate H0
ATL > IFC	0.02 ± 0.07	0.31	0.04	0.754	1	0.16	moderate H0
PTL > ATL	−0.05 ± 0.03	−1.98	0.28	0.053	0.106	0.94	anecdotal H0
PTL > IC	−0.03 ± 0.02	−1.56	0.22	0.125	0.223	0.48	anecdotal H0
PTL > IFC	−0.07 ± 0.03	−2.29	0.32	0.027	0.085	1.64	anecdotal H1
IC > ATL	0.12 ± 0.05	2.48	0.35	0.017	0.066	2.45	anecdotal H1
IC > PTL	0.11 ± 0.05	2.14	0.3	0.037	0.085	1.24	anecdotal H1
IC > IFC	0.11 ± 0.05	2.15	0.3	0.037	0.085	1.26	anecdotal H1
IFC > ATL	0.13 ± 0.04	3.06	0.43	0.004	0.028	9.24	moderate H1
IFC > PTL	0.14 ± 0.05	2.54	0.36	0.014	0.066	2.79	anecdotal H1
IFC > IC	0.16 ± 0.04	3.78	0.53	0	0.007	61.25	very strong H1

Comparison of single non-linear connectivity values between atVNS and sham condition for action planning and perception phase, using frequentist, and Bayesian *t* test. Mean difference and SEM are given for each contrast beside t-value (t), Cohen’s d (d), *p*-value (p), and FDR corrected *p*-value (p (FDR)). The Bayes factor strength of evidence label is given as evidence toward H1 if BF10 > 1 and otherwise as evidence toward H0 based on Lee and Wagenmakers.^43^

For linear connectivities, detailed results are given in Table 4. Differences between the sham and atVNS conditions were only observed in the perception phase. The connectivities of IC to IFC showed significant differences between sham and atVNS (mean difference: 0.21 ± 0.5; *p* = 0.004). Additionally, an effect of stimulation was also found for IFC to IC (mean difference: 0.15 ± 0.05; *p* = 0.04).

For non-linear connectivities, detailed results are given in Table 5. Significant differences between the sham and tVNS conditions in the action planning phase were found for the connection of PTL to IFC (mean difference: −0.10 ± 0.03). All other connectivities of the action planning phase showed no stimulation effect. For the perception phase, the non-linear connectivities of IFC to ATL differed significantly between the sham and atVNS conditions (mean difference: 0.13 ± 0.04; *p* = 0.028). Additionally, a stimulation effect was also observed for IFC to IC (mean difference: 0.16 ± 0.04; *p* = 0.007).

### Net bilateral connectivities stimulation effect

Lastly, we compared the differences of the sham versus the atVNS condition for the bilateral connectivities net effects, i.e., the differences of bilateral connectivity values (e.g., connectivity of ROI A to B minus ROI B to A). Detailed results for linear connectivities are given in Tables S2 and S3. Additionally, the sham versus tVNS condition contrasts are depicted in Figure 6B. The strength of the connectivities is depicted by the thickness of the respective arrows. Significant stimulation condition net differences are indicated by colored arrows as well as a colored text-box, whereas non-significant net connectivity differences are depicted with gray arrows. FDR correction has been applied to account for multiple testing.

There were no stimulation effects for linear connectivities in the planning phase and the perception phase. Detailed results for non-linear connectivities are given in Table S3. Significant differences of net connections between sham and atVNS condition in the action planning phase were only observed for PTL and IFC (mean difference 0.18 ± 0.05; *p* = 0.005). The asymmetry of the bilateral non-linear connectivities of PTL and IFC (IFC to PTL > PTL to IFC) was stronger in the sham condition than in the tVNS condition. In the perception phase, significant effects of sham versus atVNS condition on linear connectivities were observed between PTL and IC (mean difference: 0.14 ± 0.05; *p* = 0.008). Bilateral asymmetries (IC to PTL > PTL to IC) were stronger in the sham than in the tVNS condition. Additionally, stimulation effects on net differences were also found for PTL and IFC (mean difference 0.20 ± 0.06; *p* = 0.007). Again, bilateral asymmetries of non-linear connectivities (IFC to PTL > PTL to IFC) were stronger in the sham condition than in the atVNS condition.

## Discussion

The current study investigated the neurobiological underpinnings of intentional behavior. To this end, we used atVNS and examined its effect on action-effect integration/retrieval processes. We were interested in how atVNS impacts directed information transfer in a possibly canonical action-effect integration network driven by theta-band activity, and comprising the anterior temporal lobe (ATL), the insular cortex (IC), and the inferior frontal cortex (IFC).^8^^,^^9^

Our observations corroborate previous findings that the ATL, the IC, and the IFC are part of a canonical theta band activity-associated network for action-effect processing. However, unlike previous findings,^8^^,^^9^ also regions in the posterior parts of the temporal lobe (PTL) and the ventral visual pathway were activated during the planning and the perception phase. This was particularly evident in the active atVNS condition, providing insights into the limits of the reach and definition of ideomotor theory. Ideomotor theory proposes that the same representations are involved in the planning and perception of action effects, whereas it fails to specify the particular processes that operate on these representations.^2^^,^^3^^,^^7^ Our findings do not challenge this representational assumption, but they provide further insights into the not yet well understood processing implications, especially in view of the observation that atVNS led to the recruitment of additional brain regions, compared to sham stimulation. Together with previous findings,^9^ the current study thus establishes a foundation for a more detailed neurobiological understanding of ideomotor concepts, thereby enhancing the theoretical basis to explain intentional behavior.

While the precise neurobiological basis is unknown and many neurotransmitter systems are involved in atVNS effects,^22^^,^^31^^,^^32^^,^^33^ the catecholaminergic (NE/DA) and GABAergic systems likely play prominent roles. Notably, recent findings examined the effects of joint modulation of the NE and DA systems on action effect integration in the same experimental setup, using identical EEG analysis methods.^9^ Unlike the current study, the studied NE/DA system modulation did not account for a strong recruitment of posterior ventral stream areas. This suggests that neurobiological factors other than the NE/DA system are in effect during atVNS-induced effects on action-effect integration. This could be the GABAergic system.^27^^,^^28^^,^^29^^,^^30^ Indeed, the obtained findings are in line with the interpretation that the GABAergic system is involved in atVNS effects on action-effect integration. For this, the analysis of the directed information transfer pattern between the PTL, ATL, IC, and IFC cortical regions is of relevance. Compared to sham-stimulation, the directed information transfer was lower between cortical regions during active atVNS stimulation. This was particularly true during the perception phase, i.e., while the agent was processing the self-generated action effect. In this phase, directed information transfer from the IC to the IFC was reduced (linear and non-linear information transfer), and from the IFC to the ATL was reduced compared to sham-atVNS stimulation. This reduced information transfer suggests a relative disconnect in networked communication between cortical regions. It indicates that neural processes subserving action-effect integration become more modular because of atVNS. This relative disconnection of neural processes can be well attributed to the effects of enhanced GABAergic neural transmission, likely induced by atVNS.^27^^,^^28^^,^^29^^,^^30^^,^^32^ While the provided data cannot provide clear-cut insights that the GABAergic system is the driving force behind the observed effects, other evidence on the role of GABAergic neural transmission in networked cortical communication corroborates the interpretation that the GABAergic system is likely essential to the current pattern of findings: In the cortex, higher GABA conductance promotes shunting inhibition, which desynchronizes local assemblies and disrupts large-scale phase alignment. With weaker phase alignment, frequency-specific routing (“communication-through-coherence”)^44^ is impaired, lowering directed interactions. Drugs that enhance GABAergic activity diminish directed connectivity and cortical information flow, and these effects are most potent at low frequencies ≤12 Hz (i.e., in the theta/alpha range).^45^^,^^46^^,^^47^ Relatedly, under increased GABAergic tone (e.g., sedation), EEG/fMRI networks exhibit decreased long-range connectivity and increased local efficiency/clustering consistent with reduced inter-module directed flow in slow frequency bands.^48^^,^^49^ This is reflected in the obtained empirical signature, characterized by lower directed transfer of information between cortical regions during action-effect integration following active atVNS. Notably, the recent findings by Mayer et al.^9^ on the effects of enhanced catecholaminergic neural transmission yielded results distinct from those of the current study. This further corroborates the interpretation of atVNS-induced change of action-effect integration processes on a GABAergic basis. The more modular organization of processes in the cortical action-effect processing network may also explain why additional cortical regions in the PTL become evident during active atVNS, compared to sham-atVNS.

Critically, the observed modulations in directed neural cortical information transfer induced by atVNS provide further grounds that necessitate a more process-specific view on ideomotor principles. As mentioned above, ideomotor principles state that the same representations are involved during the planning and the perception/validation of an action effect, while the processes operating on these representations may differ. The directed connectivity findings reveal that atVNS effects were evident primarily in the perception phase of the experiment. Only there, the directed information transfer from the IC to the IFC and from the IFC to the ATL was reduced compared to sham-atVNS stimulation. This suggests that the processes involved in the perception/validation phase of the ideomotor cycle are particularly well characterized by this particular transfer, which however does not seem to play a role in the processes related to action planning.

Other processes seem to be shared by planning and perception. Note that directed information transfer works in two directions and can be expressed in changes in the strength of information transfer from brain region “A” to brain region “B” and from brain region “B“ to “A.” Thus, the symmetry/asymmetry in the directed information flow is also relevant to analyze. This analysis revealed that atVNS induced a stronger transfer of information from the IFC to PTL than vice versa, which was evident in both the planning and perception phases of the experiment.

Taken altogether, the overall pattern of directed connectivity findings shows that some, but not all, network properties in the cortical action-effect network reflect processes involved in both action-effect perception and planning, whereas other properties are more specific to one of these processes. Several reasons may explain why the PTL-IFC asymmetry is more closely tied to ideomotor principles. The PTL and the IFC may form a classical perception-action loop (ventral stream semantic/visual coding ↔ frontal motor representations). Asymmetry in directed flow here (IFC driving PTL) across both phases aligns with ideomotor theory, as it reflects the bidirectional coding of action-effects representations that remain stable across planning and perception. The PTL-IFC axis in the network may carry representational content (linking sensory features with motor intentions). Ideomotor theory is fundamentally concerned with representational overlap, aligning with this axis.^2^^,^^3^ Regarding the IC-IFC part, the insular cortex is involved in the processing of sensory information,^50^^,^^51^^,^^52^ but is also a saliency-driven region^53^ and thus less concerned with the representational aspects central for ideomotor theory. Our findings suggest a more differentiated mapping between ideomotor principles and underlying cortical network processes. The perception-specific reduction in IC-IFC connectivity under atVNS points to a role of insular-frontal interactions in validating self-produced outcomes, consistent with the insula’s role in salience and interoceptive evaluation. By contrast, the IFC-PTL asymmetry, which was modulated by atVNS yet present across both planning and perception phases, likely reflects a more stable perception-action loop linking sensory representations with frontal motor codes. Within an ideomotor framework, one may therefore distinguish (i) representational circuits that support the bidirectional coding of action-effect associations (here along the PTL-IFC axis) from (ii) evaluative/control circuits (here involving IC-IFC) that govern the contextual weighting and validation of these associations. Our data thus support a process-specific refinement of ideomotor theory, where identical representations can be subjected to distinct control operations during planning and outcome evaluation. Yet, the involvement of the posterior temporal/ventral-stream region (PTL) should be regarded as an exploratory, data-driven observation. The PTL cluster emerged from the beamformer analysis rather than from *a-priori* hypothesis. While the PTL findings are compatible with known ventral-stream contributions to perceptual components of action-effect integration, they require replication in future work.

The study advances the neurobiological understanding of intentional behavior by combining atVNS with analyses of directed cortical information flow in a canonical action-effect integration network. While confirming that the ATL, IC, and IFC form a theta-associated core network, it is demonstrated that atVNS recruits additional posterior temporal/ventral visual regions and increases the segregation of neural processes within the network. This pattern is most consistent with GABAergic modulation, distinguishing the present findings on catecholaminergic contributions to action-effect integration. The findings demonstrate that ideomotor principles, formulated initially in purely cognitive terms and with respect to representations, would strongly benefit from refinement regarding the involved neurophysiological processes: some aspects of the network (e.g., IFC-PTL asymmetry) are involved in both action-effect perception and planning, whereas others (e.g., IC-IFC connectivity) are specific for one of these processes. The study provides nuanced grounds to explain how intentional behavior emerges from dynamic cortical interactions and links ideomotor theory with neurobiological mechanisms of action control.

### Limitations of the study

While atVNS is a promising non-invasive brain stimulation tool to probe neuromodulatory influences on cortical network dynamics, its precise mechanisms remain incompletely understood. Although converging evidence suggests a role for the GABAergic system, other neurotransmitter systems may also contribute to the observed effects.

Although participants could guess the stimulation condition above chance, several features of the results indicate that expectancy is unlikely to account for the stimulation-related effects. Expectancy-driven neural changes typically manifest as broad shifts in arousal or attention, whereas our effects were connection- and phase-specific, confined to directed connectivity. Connectivity did not differ between participants who correctly vs. incorrectly guessed the active condition, and the one significant comparison was consistent with chance and unrelated to the reported stimulation effects. Thus, while incomplete blinding remains a limitation, the specificity and anatomical plausibility of the reported connectivity changes make an expectancy-driven explanation unlikely.

## Resource availability

### Lead contact

Further information and requests for resources and reagents should be directed to and will be fulfilled by the lead contact, Christian Beste (christian.beste@ukdd.de).

### Materials availability

This study did not generate new unique reagents or other materials.

### Data and code availability

Behavioral and EEG raw data have been deposited at zenodo.org and are publicly available at https://doi.org/10.5281/zenodo.17107938.
All original code has been deposited at OSF and is publicly available at https://doi.org/10.5281/zenodo.17107938. Any additional information required to reanalyze the data reported in this article is available from the lead contact upon request.

## Acknowledgments

This work was supported by Grants from the 10.13039/501100001659Deutsche Forschungsgemeinschaft (DFG) FOR 2790 and BE4045/65-1.

## Author contributions

Conceptualization: C.B.; formal analysis: M.M.; funding acquisition: C.B. and B.H.; investigation: J.M.; methodology: M.M., B.H., and C.B.; project administration: C.B.; resources: C.B.; software: M.M.; supervision: C.B.; validation: C.B.; visualization: M.M.; writing – original draft and preparation: M.M. and C.B.; writing – review and editing: all authors. All of the authors reviewed and approved the article for publication.

## Declaration of interests

The authors declare no competing interests.

## STAR★Methods

### Key resources table

**Table:** 

REAGENT or RESOURCE	SOURCE	IDENTIFIER
**Deposited data**		

Raw data behavior	This paper	https://doi.org/10.5281/zenodo.17107938.
Raw data EEG	This paper	https://doi.org/10.5281/zenodo.17107938.
Original Code	This paper	https://doi.org/10.5281/zenodo.17107938.

**Software and algorithms**		

MATLAB	https://de.mathworks.com/products/matlab.html	RRID:SCR_001622
BrainVision Recorder	https://www.brainproducts.com/productdetails.php?id=21	RRID:SCR_016331
EEGLAB	http://sccn.ucsd.edu/eeglab/index.html	RRID:SCR_007292
FieldTrip	https://www.fieldtriptoolbox.org	RRID:SCR_004849
Presentation	http://www.neurobs.com/	RRID:SCR_002521

### Experimental model and study participant details

#### Sample

N=52 healthy human participants were recruited. Two participants did not participate in both recording sessions, resulting in a final sample of 50 participants (19–35 years, M = 26, SD = 3.95; 24 male, 26 females). The effects of biological sex were not examined in the study, which is a limitation. All had normal or corrected-to-normal vision and no history of neurological/psychiatric disorders, substance abuse, brain surgery, tumors, intracranial metal implants, or psychoactive medication. Additionally, atVNS criteria excluded pregnancy, seizure/migraine susceptibility, and implanted devices. Participants provided written informed consent and received 35 EUR as compensation. The study was approved by the Ethics Committee of the Medical Faculty at TU Dresden and was conducted in accordance with the Declaration of Helsinki.

### Method details

#### Task

The task used was identical to that in previous studies,^8^^,^^9^ which is also a prerequisite for comparing the effects of atVNS with those of MPH. The paradigm was modified from Dignath et al.,^36^ which itself builds on the action-effect framework described by Elsner and Hommel.^54^ Our implementation is illustrated in Figure 1. The experiment was performed using Presentation software (Version 21.1, Neurobehavioral Systems, Inc., Berkeley, CA, www.neurobs.com). Participants were seated in front of a 24-inch TFT.

To facilitate the acquisition of action-effect contingencies, participants first completed 30 practice trials before the main task, which consisted of 300 trials. Each trial began with a central grey fixation point displayed for 2000 ms, followed by a color change to either red or blue, serving as the cue stimulus. This cue remained on screen for an additional 2,000 ms. Unlike the timing used by Dignath et al.,^36^ participants in our study responded after the cue had disappeared, using either the left or right CTRL key on a QWERTZ keyboard. Response mappings were counterbalanced across participants: half were instructed to press the left key for blue and the correct key for red, while the remaining half received the opposite instruction. Correct responses immediately triggered two sensory action-effects: a visual stimulus (either a flickering image of a sheep or a cat at 4.5 or 8 Hz, respectively, displayed for 2000 ms) and a corresponding auditory stimulus (the sound of a sheep or cat, played for 700 ms at approximately 55 dB). These visual flickers were designed to elicit steady-state visual evoked potentials (SSVEPs) at the respective frequencies. Thus, the action effect is determined on a neurophysiological level. The EEG frequency-tagging approach used here leverages the brain's capacity to entrain to external rhythmic stimulation—such as flickering visual input—enabling us to associate specific neural responses with learned action effects. After participants had acquired the action-effect associations, we observed increased neural activity at the frequency previously linked to a particular effect, even before the action was executed.^8^^,^^9^ This suggests that the corresponding action-effect was reactivated in anticipation, reflecting preparatory neural processes. Incorrect, premature, or delayed responses (>1000 ms) did not trigger action-effects but instead led to feedback indicating a timing or accuracy error. The assignment of cue colors, response keys, and associated action-effects (sheep/cat) was counterbalanced across participants, resulting in eight distinct conditions. Trials were randomized within each condition, resulting in an approximately equal distribution of the two flicker frequencies (approximately 150 trials per frequency per participant).

#### atVNS procedure and blinding of participants

The study employed a sham-controlled crossover design, with participants blinded to stimulation type. Each participant completed two sessions, spaced at least one week apart to reduce carryover effects, with most completing both within a month. Session order (active–sham vs. sham–active) was counterbalanced across participants and participants were randomly assigned to the groups with the specific session order. Following earlier protocols,^32^^,^^55^ stimulation started approximately 20 minutes before task onset and continued for the entire task period. Stimulation was delivered with the atVNS nextGen research device (tVNS Technologies). For intensity adjustment, participants underwent a series of ascending and descending 10-s trials, starting at 0.1 mA and increasing in 0.2 mA steps until a rating of 8 on a 10-point scale (0 = no sensation; 3 = light tingling; 6 = strong tingling; 10 = painful) was reached, followed by descending trials until ratings fell to ≤6. This procedure was repeated twice, and the final intensity was set as the meaning of four selected values. In the active condition, electrodes were positioned at the cymba conchae, a region with vagal innervation. In contrast, in the sham condition, electrodes were placed on the earlobe, which lacks vagal fibers and does not induce brainstem or cortical activation.^56^^,^^57^ To ensure blinding, stimulation was applied in both sessions. Parameters were set according to established recommendations^31^: continuous stimulation at 25 Hz, with a pulse width of 200–300 ms, applied exclusively to the left ear to minimize potential cardiac effects.^58^^,^^59^ At the end of each session, participants completed a side-effects questionnaire, rating possible adverse sensations (e.g., headache, neck pain, nausea, facial muscle contractions, stinging under the electrodes, refer to Table 1) on a 5-point scale. After completing both sessions, they were asked to indicate which session they believed involved active stimulation.

#### EEG recording and pre-processing

EEG recordings were obtained with QuickAmp and BrainAmp amplifiers (Brain Products GmbH, Gilching, Germany) using 60 equidistantly placed Ag–AgCl electrodes. Data were sampled at 500 Hz, referenced to Fpz, with electrode impedances kept below 5 kΩ. Preprocessing was performed in accordance with previous studies^8^^,^^9^ using Matlab 2021b (The MathWorks Corp.) and Automagic^60^ and EEGLAB.^61^ Signals were first downsampled to 256 Hz, and flat EOG channels were interpolated. Subsequent steps included the PREP pipeline^62^ and EEGLAB’s clean_rawdata() routine. PREP attenuates 50 Hz line noise via a multitaper approach and applies robust average referencing after discarding contaminated channels. The clean_rawdata() procedure detrended the signal using a 0.5 Hz FIR high-pass filter (order 1286, stop-band attenuation of 80 dB, and a transition band of 0.25–0.75 Hz), and identified flat, noisy, or outlier channels for removal. Artifact Subspace Reconstruction (ASR)^63^ was then applied with a burst criterion of 15, reconstructing abnormal epochs (>15 SD above calibration data); other segments were discarded. A low-pass filter at 40 Hz (sinc FIR, order 86)^64^ was subsequently applied. Ocular artifacts were corrected by the subtraction method.^65^ Independent component analysis (ICA) combined with MARA^66^^,^^67^ automatically identified and removed components related to blinks, muscular activity, or residual eye movements. Components classified as cardiac in origin were detected via ICLabel (threshold = 0.8) and removed. Finally, excluded channels were interpolated (spherical interpolation).

#### Time-frequency decomposition and EEG beamforming analyses

EEG data segmentation was carried out in Brain Vision Analyzer (v2.1, Brain Products GmbH, Gilching, Germany). Epochs were defined relative to (i) cue onset (−2000 to +4000 ms; cue-locked) and (ii) the response or action effect onset (−2000 to +4000 ms; action effect-locked), separately for both flicker frequencies (4.5 Hz and 8 Hz). As done in previous studies, the 8 Hz flicker frequency served as a control condition outside the theta range, allowing contrasts with 4.5 Hz flicker stimulation to disentangle theta-specific entrainment from general frequency-tagging effects, thereby isolating neural mechanisms underlying action–effect binding (AEB).^8^^,^^9^ The following analysis steps were performed in Matlab using the FieldTrip Toolbox.^68^ Time–frequency decomposition of both cue-locked and action effect-locked segments was conducted with Morlet wavelets (5 cycles; wavelet length = 3). To determine relative power changes, baseline normalization was applied by referencing to a 200 ms pre-stimulus interval (−350 to −150 ms). Mean baseline power was subtracted from each data point, and the difference was divided by the baseline mean to obtain relative power changes. Two temporal phases were defined: the *action planning* phase (0–1000 ms cue-locked), and the *perception* phase (0–1000 ms action effect-locked). Each phase was assumed to capture distinct cognitive operations.

Source localization for the two focused phases of the experiment was performed using Dynamic Imaging of Coherent Sources (DICS) beamforming.^69^ A forward model based on the Montreal Neurological Institute (MNI) template brain^70^ enabled projection of EEG signals into source space on an evenly spaced grid.^68^ Spectral power and cross-spectral density matrices were estimated with a Hanning taper in the 4–5 Hz range. After co-registering electrode positions with the head model, the brain volume was divided into a 5 mm resolution grid, and the leadfield matrix was computed for each voxel. A common spatial filter was then applied across phases with a 5% regularization parameter. To correct for center bias, source estimates were normalized against spatially inhomogeneous noise, calculated from the smallest eigenvalue of the cross-spectral density matrix.^71^

#### Directed connectivity analysis

The basis for the directed connectivity analysis was the results of the DICS beamforming step, which we used to define regions of interest (ROIs). For this, first, voxels located within 3% of the local maxima of source activity inside relevant neuroanatomical regions were identified. These voxels were then subjected to clustering using the Density-Based Spatial Clustering of Applications with Noise (DBSCAN) algorithm.^72^^,^^73^ To capture neighboring voxels effectively, the epsilon parameter in the DBSCAN algorithm was set to twice the voxel edge length. Clusters obtained in this way were linked to anatomical labels provided by the Automated Anatomical Labeling (AAL) atlas,^40^ forming the basis for ROI selection for each phase (i.e., Action Planning and Perception). For every voxel within these ROIs and phases, virtual neural time series were reconstructed using a Linearly Constrained Minimum Variance (LCMV) beamformer.^71^ The voxel-wise signals were obtained by applying the LCMV spatial filter to the time-domain data. These reconstructed time series were then subjected to time–frequency decomposition with Morlet wavelets centered at 4.5 Hz. Finally, power estimates were averaged across voxels within each ROI and phase.

The directed connectivity analysis was performed using nCREANN (nonlinear Causal Relationship Estimation by Artificial Neural Network), previously established in our laboratory.^37^^,^^41^^,^^74^^,^^75^ The method extends the nonlinear Multivariate Autoregressive (MVAR) framework, in which neural activity within a given ROI is modeled as a function of past activity in multiple regions. The nCREANN model explicitly incorporates both linear and nonlinear components to capture these dynamics. Lagged neural signals serve as predictors for future samples, enabling the reconstruction of directed interactions across brain areas. The nCREANN model is represented by the equation:

(Equation 1) $$x\left(n\right)=f\left(x_{p}\right)+\sigma \left(n\right)$$

where $x_{p}={\left[x_{1}\left(n-1\right),x_{2}\left(n-1\right),\cdots ,x_{M}\left(n-p\right)\;\right]}^{T}$ denotes the vector of *p* previous samples of *M* time series and $\sigma \left(n\right)={\left[\sigma_{1},\sigma_{2},\ldots ,\sigma_{M}\;\right]}^{T}$ represents the residual of the model.

nCREANN models linear and nonlinear aspects of information exchange between brain regions. Prior work has emphasized that combining linear and nonlinear perspectives yields a more complete account of large-scale neurodynamics. In nCREANN, the nonlinear Multivariate Autoregressive (nMVAR) model is implemented using a single-hidden-layer feed-forward neural network. This network employs nonlinear activation functions for hidden neurons and linear functions at the output layer. To extract both linear and nonlinear information embedded within the network's structure, the linear (***f***^*Lin*^) and nonlinear ***f***^*NonLin*^ parts of the network input-output mapping ***f***(.) Are separated:

(Equation 2) $$f=f^{Lin}+f^{NonLin}$$

This separation is achieved by dividing the linear and nonlinear parts of the hidden neurons’ functions assessed by their corresponding Taylor expansion. To compute linear effective connectivity $\left({lC}_{i\to j}\right)$, the approach involves ***f***^*Lin*^ and is determining it by multiplying the network's connection weights with the scaling parameters of the hidden neurons. This measure signifies the degree to which the *i*^th^ input node linearly influences the *j*^th^ output neuron. Nonlinear effective connectivity (${NC}_{i\to j}$) from *x*_*i*_ to *x*_*j*_is defined as the ratio of the network's estimation errors:

(Equation 3) $${NC}_{i\to j}=\ln\left(\frac{\left\langle {{\left(\epsilon_{j}\right)}_{x_{i}\_Lin}}^{2}\right\rangle }{\left\langle {\left(\epsilon_{j}\right)}^{2}\right\rangle }\right)$$

Here, the numerator's estimation error pertains to scenarios where *x*_*i*_ contributes to *x*_*j*_is defined purely linearly, while the denominator's error corresponds to situations where all input signals exert both linear and nonlinear effects on *x*_*j*_. ${NC}_{i\to j}$ quantifies the extent of the nonlinear causal effect of *x*_*i*_ on *x*_*j*_. The model order was optimized using the Schwarz Bayesian Criterion, yielding an order of 20 for adults and 8 for adolescents, as implemented in the ARfit toolbox for Multivariate Autoregressive Model Fitting.^76^ For nonlinear modeling, a multilayer perceptron (MLP) neural network with a single hidden layer and six hidden neurons was trained using gradient descent back-propagation with momentum (*α*) and an adaptive learning rate (*η*). Generalization was ensured by early stopping and a five-fold permuted cross-validation, with data split into 80% training, 10% validation, and 10% testing sets. The network operated in incremental mode with randomly initialized weights in the range −0.5 to 0.5.

Connectivity maps were projected onto a head model, where directed arrows represented information flow between clusters of sources, and arrow thickness corresponded to connection strength. Model performance was evaluated using mean square error (MSE) and the coefficient of determination (R^2^) for both training and test data. Comparable R^2^ values across training and testing indicated reliable generalization, with R^2^ values ranging from 0 (poor fit) to 1 (perfect fit). To test statistical significance, connectivity estimates were compared against surrogate distributions generated by a time-shifted randomization approach with 100 surrogate datasets. Identical network parameters were applied to both original and surrogate data to ensure robust comparisons.

### Quantification and statistical analysis

If not otherwise stated, mean values and the standard error of the mean (SE) are reported. Behavioral differences (mean RTs and accuracy) between sham and atVNS active stimulation condition were assessed using paired-sample t-tests, with accompanying Bayes factors (BF_10_). BF_10_ values were interpreted following Lee and Wagenmakers^43^: evidence for H1: anecdotal (1–3), moderate (3–10), strong (10–30), very strong (30–100), extreme (>100); evidence for H0: anecdotal (.33–1), moderate (.1–.33), strong (.03–.1), very strong (.01–.03), extreme (<.01). Unless otherwise stated, α was set at .05.

To examine the modulatory effect of action effect flicker frequencies, average power values during the perception phase were compared across frequencies using repeated-measures ANOVAs, and relative power changes were tested with paired-sample t-tests. Multiple comparisons were controlled using FDR correction (α = .05).^39^

Connectivity analyses were conducted as follows: First, asymmetries between ROIs were tested with paired-sample t-tests on bidirectional connection pairs (FDR-corrected, α = .05), with BF_10_ additionally computed. Second, single connectivities were compared between stimulation conditions, i.e. sham versus atVNS condition. Third, bilateral net differences were compared between sham and atVNS condition. Net differences were determined by subtracting the respective bilateral connectivities (e.g. connectivity of ROI A to B minus ROI B to A). For both single connectivity contrasts as well as net difference contrasts, paired-sample and Bayesian t-tests were used, and p values were corrected using FDR.

## References

[1] Hommel B., Müsseler J., Aschersleben G., Prinz W. (2001). The Theory of Event Coding (TEC): a framework for perception and action planning. Behav. Brain Sci..

[2] Shin Y.K., Proctor R.W., Capaldi E.J. (2010). A review of contemporary ideomotor theory. Psychol. Bull..

[3] Waszak F., Cardoso-Leite P., Hughes G. (2012). Action effect anticipation: neurophysiological basis and functional consequences. Neurosci. Biobehav. Rev..

[4] Kühn S., Keizer A.W., Colzato L.S., Rombouts S.A.R.B., Hommel B. (2011). The neural underpinnings of event-file management: evidence for stimulus-induced activation of and competition among stimulus-response bindings. J. Cogn. Neurosci..

[5] Elsner B., Hommel B., Mentschel C., Drzezga A., Prinz W., Conrad B., Siebner H. (2002). Linking actions and their perceivable consequences in the human brain. Neuroimage.

[6] Melcher D., Colby C.L. (2008). Trans-saccadic perception. Trends Cogn. Sci..

[7] Melcher T., Winter D., Hommel B., Pfister R., Dechent P., Gruber O. (2013). The neural substrate of the ideomotor principle revisited: Evidence for asymmetries in action-effect learning. Neuroscience.

[8] Mayer J., Mückschel M., Talebi N., Hommel B., Beste C. (2025). Directed connectivity in theta networks supports action-effect integration. Neuroimage.

[9] Mayer J., Helin Koyun A., Mückschel M., Roessner V., Hommel B., Beste C. (2025). Pharmacological modulation of directed network communication and neural hubs in action–effect integration. Int. J. Neuropsychopharmacol..

[10] Mayer J., Mückschel M., Hommel B., Beste C. (2025). Maturational Changes in Action-Effect Integration Processes Are Reflected by Changes in the Directed Cortical Network Communication. Hum. Brain Mapp..

[11] Faraone S.V. (2018). The pharmacology of amphetamine and methylphenidate: Relevance to the neurobiology of attention-deficit/hyperactivity disorder and other psychiatric comorbidities. Neurosci. Biobehav. Rev..

[12] Aston-Jones G., Cohen J.D. (2005). An integrative theory of locus coeruleus-norepinephrine function: adaptive gain and optimal performance. Annu. Rev. Neurosci..

[13] Hauser T.U., Fiore V.G., Moutoussis M., Dolan R.J. (2016). Computational Psychiatry of ADHD: Neural Gain Impairments across Marrian Levels of Analysis. Trends Neurosci..

[14] Salinas E., Sejnowski T.J. (2001). Gain modulation in the central nervous system: where behavior, neurophysiology, and computation meet. Neuroscientist.

[15] Lew S.E., Tseng K.Y. (2014). Dopamine modulation of GABAergic function enables network stability and input selectivity for sustaining working memory in a computational model of the prefrontal cortex. Neuropsychopharmacology.

[16] Gao Y., Koyun A.H., Roessner V., Stock A.-K., Mückschel M., Colzato L., Hommel B., Beste C. (2025). Transcranial direct current stimulation and methylphenidate interact to increase cognitive persistence as a core component of metacontrol: Evidence from aperiodic activity analyses. Brain Stimul..

[17] Koyun A.H., Werner A., Kuntke P., Roessner V., Beste C., Stock A.-K. (2025). GABA and Glx levels in cortico-subcortical networks predict catecholaminergic effects on response inhibition. J. Psychopharmacol..

[18] Salgado H., Garcia-Oscos F., Martinolich L., Hall S., Restom R., Tseng K.Y., Atzori M. (2012). Pre- and postsynaptic effects of norepinephrine on γ-aminobutyric acid-mediated synaptic transmission in layer 2/3 of the rat auditory cortex. Synapse.

[19] Trantham-Davidson H., Neely L.C., Lavin A., Seamans J.K. (2004). Mechanisms Underlying Differential D1 versus D2 Dopamine Receptor Regulation of Inhibition in Prefrontal Cortex. J. Neurosci..

[20] Tremblay R., Lee S., Rudy B. (2016). GABAergic Interneurons in the Neocortex: From Cellular Properties to Circuits. Neuron.

[21] Bryson A., Hatch R.J., Zandt B.-J., Rossert C., Berkovic S.F., Reid C.A., Grayden D.B., Hill S.L., Petrou S. (2020). GABA-mediated tonic inhibition differentially modulates gain in functional subtypes of cortical interneurons. Proc. Natl. Acad. Sci. USA.

[22] Colzato L., Beste C. (2020). A literature review on the neurophysiological underpinnings and cognitive effects of transcutaneous vagus nerve stimulation: challenges and future directions. J. Neurophysiol..

[23] Fischer R., Ventura-Bort C., Hamm A., Weymar M. (2018). Transcutaneous vagus nerve stimulation (tVNS) enhances conflict-triggered adjustment of cognitive control. Cogn. Affect. Behav. Neurosci..

[24] Giraudier M., Ventura-Bort C., Burger A.M., Claes N., D’Agostini M., Fischer R., Franssen M., Kaess M., Koenig J., Liepelt R. (2022). Evidence for a modulating effect of transcutaneous auricular vagus nerve stimulation (taVNS) on salivary alpha-amylase as indirect noradrenergic marker: A pooled mega-analysis. Brain Stimul..

[25] Ventura-Bort C., Wirkner J., Genheimer H., Wendt J., Hamm A.O., Weymar M. (2018). Effects of Transcutaneous Vagus Nerve Stimulation (tVNS) on the P300 and Alpha-Amylase Level: A Pilot Study. Front. Hum. Neurosci..

[26] Warren C.M., Tona K.D., Ouwerkerk L., van Paridon J., Poletiek F., van Steenbergen H., Bosch J.A., Nieuwenhuis S. (2019). The neuromodulatory and hormonal effects of transcutaneous vagus nerve stimulation as evidenced by salivary alpha amylase, salivary cortisol, pupil diameter, and the P3 event-related potential. Brain Stimul..

[27] Dietrich S., Smith J., Scherzinger C., Hofmann-Preiß K., Freitag T., Eisenkolb A., Ringler R. (2008). A novel transcutaneous vagus nerve stimulation leads to brainstem and cerebral activations measured by functional MRI Funktionelle Magnetresonanztomographie zeigt Aktivierungen des Hirnstamms und weiterer zerebraler Strukturen unter transkutaner Vagusnervstimulation. Biomed. Tech..

[28] Frangos E., Ellrich J., Komisaruk B.R. (2015). Non-invasive access to the vagus nerve central projections via electrical stimulation of the external ear: fMRI evidence in humans. Brain Stimul..

[29] Hein E., Nowak M., Kiess O., Biermann T., Bayerlein K., Kornhuber J., Kraus T. (2013). Auricular transcutaneous electrical nerve stimulation in depressed patients: a randomized controlled pilot study. J. Neural Transm..

[30] Yakunina N., Kim S.S., Nam E.-C. (2017). Optimization of Transcutaneous Vagus Nerve Stimulation Using Functional MRI. Neuromodulation.

[31] Farmer A.D., Strzelczyk A., Finisguerra A., Gourine A.V., Gharabaghi A., Hasan A., Burger A.M., Jaramillo A.M., Mertens A., Majid A. (2020). International Consensus Based Review and Recommendations for Minimum Reporting Standards in Research on Transcutaneous Vagus Nerve Stimulation (Version 2020). Front. Hum. Neurosci..

[32] Konjusha A., Yu S., Mückschel M., Colzato L., Ziemssen T., Beste C. (2023). Auricular Transcutaneous Vagus Nerve Stimulation Specifically Enhances Working Memory Gate Closing Mechanism: A System Neurophysiological Study. J. Neurosci..

[33] Ludwig M., Wienke C., Betts M.J., Zaehle T., Hämmerer D. (2021). Current challenges in reliably targeting the noradrenergic locus coeruleus using transcutaneous auricular vagus nerve stimulation (taVNS). Auton. Neurosci..

[34] Beste C., Moll C.K.E., Pötter-Nerger M., Münchau A. (2018). Striatal Microstructure and Its Relevance for Cognitive Control. Trends Cogn. Sci. (Regul. Ed.).

[35] Koyun A.H., Talebi N., Werner A., Wendiggensen P., Kuntke P., Roessner V., Beste C., Stock A.-K. (2024). Interactions of catecholamines and GABA+ in cognitive control: Insights from EEG and 1H-MRS. Neuroimage.

[36] Dignath D., Kiesel A., Frings C., Pastötter B. (2020). Electrophysiological evidence for action-effect prediction. J. Exp. Psychol. Gen..

[37] Talebi N., Nasrabadi A.M., Mohammad-Rezazadeh I., Coben R. (2019). nCREANN: Nonlinear Causal Relationship Estimation by Artificial Neural Network; Applied for Autism Connectivity Study. IEEE Trans. Med. Imaging.

[38] Benjamini Y., Hochberg Y. (1995). Controlling the False Discovery Rate: A Practical and Powerful Approach to Multiple Testing. J. Roy. Stat. Soc. B.

[39] Genovese C.R., Lazar N.A., Nichols T. (2002). Thresholding of Statistical Maps in Functional Neuroimaging Using the False Discovery Rate. Neuroimage.

[40] Rolls E.T., Huang C.-C., Lin C.-P., Feng J., Joliot M. (2020). Automated anatomical labelling atlas 3. Neuroimage.

[41] Elmers J., Mückschel M., Akgün K., Ziemssen T., Beste C. (2025). Variations in neuronal cytoskeletal integrity affect directed communication in distributed networks during inhibitory control. Commun. Biol..

[42] Ghorbani F., Zhou X., Talebi N., Roessner V., Hommel B., Prochnow A., Beste C. (2024). Neural connectivity patterns explain why adolescents perceive the world as moving slow. Commun. Biol..

[43] Lee M.D., Wagenmakers E.-J. (2014).

[44] Fries P. (2015). Rhythms for Cognition: Communication through Coherence. Neuron.

[45] Chen Y., Li S., Wu F., Zou L., Zhang J. (2022). Altered functional and directed connectivity in propofol-induced loss of consciousness: A source-space resting-state EEG study. Clin. Neurophysiol..

[46] Maksimow A., Silfverhuth M., Långsjö J., Kaskinoro K., Georgiadis S., Jääskeläinen S., Scheinin H. (2014). Directional Connectivity between Frontal and Posterior Brain Regions Is Altered with Increasing Concentrations of Propofol. PLoS One.

[47] Pullon R.M., Yan L., Sleigh J.W., Warnaby C.E. (2020). Granger Causality of the Electroencephalogram Reveals Abrupt Global Loss of Cortical Information Flow during Propofol-induced Loss of Responsiveness. Anesthesiology.

[48] Boly M., Moran R., Murphy M., Boveroux P., Bruno M.-A., Noirhomme Q., Ledoux D., Bonhomme V., Brichant J.-F., Tononi G. (2012). Connectivity Changes Underlying Spectral EEG Changes during Propofol-Induced Loss of Consciousness. J. Neurosci..

[49] Sattin D., Duran D., Visintini S., Schiaffi E., Panzica F., Carozzi C., Rossi Sebastiano D., Visani E., Tobaldini E., Carandina A. (2021). Analyzing the Loss and the Recovery of Consciousness: Functional Connectivity Patterns and Changes in Heart Rate Variability During Propofol-Induced Anesthesia. Front. Syst. Neurosci..

[50] Cauda F., Costa T., Torta D.M.E., Sacco K., D’Agata F., Duca S., Geminiani G., Fox P.T., Vercelli A. (2012). Meta-analytic clustering of the insular cortex. Neuroimage.

[51] Droutman V., Bechara A., Read S.J. (2015). Roles of the Different Sub-Regions of the Insular Cortex in Various Phases of the Decision-Making Process. Front. Behav. Neurosci..

[52] Gogolla N. (2017). The insular cortex. Curr. Biol..

[53] Uddin L.Q. (2015). Salience processing and insular cortical function and dysfunction. Nat. Rev. Neurosci..

[54] Elsner B., Hommel B. (2001). Effect anticipation and action control. J. Exp. Psychol. Hum. Percept. Perform..

[55] Beste C., Steenbergen L., Sellaro R., Grigoriadou S., Zhang R., Chmielewski W., Stock A.-K., Colzato L. (2016). Effects of Concomitant Stimulation of the GABAergic and Norepinephrine System on Inhibitory Control - A Study Using Transcutaneous Vagus Nerve Stimulation. Brain Stimul..

[56] Kraus T., Kiess O., Hösl K., Terekhin P., Kornhuber J., Forster C. (2013). CNS BOLD fMRI Effects of Sham-Controlled Transcutaneous Electrical Nerve Stimulation in the Left Outer Auditory Canal – A Pilot Study. Brain Stimul..

[57] Peuker E.T., Filler T.J. (2002). The nerve supply of the human auricle. Clin. Anat..

[58] Kreuzer P.M., Landgrebe M., Husser O., Resch M., Schecklmann M., Geisreiter F., Poeppl T.B., Prasser S.J., Hajak G., Langguth B. (2012). Transcutaneous Vagus Nerve Stimulation: Retrospective Assessment of Cardiac Safety in a Pilot Study. Front. Psychiatr..

[59] Nemeroff C.B., Mayberg H.S., Krahl S.E., McNamara J., Frazer A., Henry T.R., George M.S., Charney D.S., Brannan S.K. (2006). VNS Therapy in Treatment-Resistant Depression: Clinical Evidence and Putative Neurobiological Mechanisms. Neuropsychopharmacology.

[60] Pedroni A., Bahreini A., Langer N. (2019). Automagic: Standardized preprocessing of big EEG data. Neuroimage.

[61] Delorme A., Makeig S. (2004). EEGLAB: an open source toolbox for analysis of single-trial EEG dynamics including independent component analysis. J. Neurosci. Methods.

[62] Bigdely-Shamlo N., Mullen T., Kothe C., Su K.-M., Robbins K.A. (2015). The PREP pipeline: standardized preprocessing for large-scale EEG analysis. Front. Neuroinf..

[63] Mullen T., Kothe C., Chi Y.M., Ojeda A., Kerth T., Makeig S., Cauwenberghs G., Jung T.-P. (2013). Real-Time Modeling and 3D Visualization of Source Dynamics and Connectivity Using Wearable EEG. Conf. Proc. IEEE Eng. Med. Biol. Soc..

[64] Widmann A., Schröger E., Maess B. (2015). Digital filter design for electrophysiological data – a practical approach. J. Neurosci. Methods.

[65] Parra L.C., Spence C.D., Gerson A.D., Sajda P. (2005). Recipes for the linear analysis of EEG. Neuroimage.

[66] Winkler I., Haufe S., Tangermann M. (2011). Automatic Classification of Artifactual ICA-Components for Artifact Removal in EEG Signals. Behav. Brain Funct..

[67] Winkler I., Brandl S., Horn F., Waldburger E., Allefeld C., Tangermann M. (2014). Robust artifactual independent component classification for BCI practitioners. J. Neural. Eng..

[68] Oostenveld R., Fries P., Maris E., Schoffelen J.-M. (2011). FieldTrip: Open Source Software for Advanced Analysis of MEG, EEG, and Invasive Electrophysiological Data. Comput. Intell. Neurosci..

[69] Gross J., Kujala J., Hämäläinen M., Timmermann L., Schnitzler A., Salmelin R. (2001). Dynamic imaging of coherent sources: Studying neural interactions in the human brain. Proc. Natl. Acad. Sci. USA.

[70] Collins D.L., Zijdenbos A.P., Kollokian V., Sled J.G., Kabani N.J., Holmes C.J., Evans A.C. (1998). Design and construction of a realistic digital brain phantom. IEEE Trans. Med. Imaging.

[71] Van Veen B.D., Van Drongelen W., Yuchtman M., Suzuki A. (1997). Localization of brain electrical activity via linearly constrained minimum variance spatial filtering. IEEE Trans. Biomed. Eng..

[72] Adelhöfer N., Schreiter M.L., Beste C. (2020). Cardiac cycle gated cognitive-emotional control in superior frontal cortices. Neuroimage.

[73] Ester M., Kriegel H.-P., Sander J., Xu X. (1996). Proceedings of the Second International Conference on Knowledge Discovery and Data Mining KDD’96.

[74] Elmers J., Yu S., Talebi N., Prochnow A., Beste C. (2024). Neurophysiological effective network connectivity supports a threshold-dependent management of dynamic working memory gating. iScience.

[75] Talebi N., Prochnow A., Frings C., Münchau A., Mückschel M., Beste C. (2024). Neural mechanisms of adaptive behavior: Dissociating local cortical modulations and interregional communication patterns. iScience.

[76] Neumaier A., Schneider T. (2001). Estimation of parameters and eigenmodes of multivariate autoregressive models. ACM Trans. Math Software.

