# Supplementary material for: Auricular transcutaneous vagus nerve stimulation alters directed cortical communication during intentional actions
Source: iScience. 2025 Dec 29;29(2):114571. doi: 10.1016/j.isci.2025.114571 (PMC12828603; doi:10.1016/j.isci.2025.114571)
Supplement: Document S1. Tables S1–S3 [file mmc1.pdf]

iScience, Volume *29*

Supplemental information

Auricular transcutaneous vagus nerve stimulation alters directed cortical communication during intentional actions

Moritz Mückschel, Jasmin Mayer, Bernhard Hommel, and Christian Beste

Supplemental Table S1

Linear connectivities blinding comparison, related to Table 1

Group comparison of linear connectivities for each phase and condition between participants who failed to correctly estimate the time point of active stimulation (n=33) and those who did not estimate correctly (n=15), using two-sample t-tests. All p-values were FDR corrected.

Connectivity	linear							
	sham				tVNS			
	planning		perception		planning		perception	
	p	*p (FDR)*	*p*	*p (FDR)*	*p*	*p (FDR)*	*p*	*p (FDR)*
PTL > ATL	0.565	0.716	0.244	0.928	0.532	0.581	0.425	0.797
IC > ATL	0.221	0.716	0.871	0.928	0.094	0.355	0.845	0.894
IFC > ATL	0.351	0.716	0.358	0.928	0.635	0.635	0.465	0.797
ATL > PTL	0.581	0.716	0.810	0.928	0.148	0.355	0.458	0.797
IC > PTL	0.125	0.716	0.361	0.928	0.395	0.474	0.894	0.894
IFC > PTL	0.505	0.716	0.928	0.928	0.381	0.474	0.176	0.747
ATL > IC	0.087	0.716	0.801	0.928	0.300	0.474	0.698	0.894
PTL > IC	0.344	0.716	0.577	0.928	0.123	0.355	0.249	0.747
IFC > IC	0.597	0.716	0.858	0.928	0.318	0.474	0.624	0.894
ATL > IFC	0.861	0.861	0.195	0.928	0.018	0.220	0.160	0.747
PTL > IFC	0.441	0.716	0.821	0.928	0.266	0.474	0.205	0.747
IC > IFC	0.767	0.836	0.441	0.928	0.143	0.355	0.776	0.894

Supplemental Table S2

Contrasts of bilateral differences of linear connectivities, related to Results

Comparison of bilateral differences of linear connectivities values between tVNS and sham condition for action planning and perception phase, using frequentist and Bayesian t-test. Mean difference and SEM are given for each contrast beside t-value (t), Cohen's d (d), p-value (p) and FDR corrected p-value (p (FDR)). The Bayes factor strength of evidence label is given as evidence towards H1 if BF10 > 1 and otherwise as evidence towards H0 based on Lee and Wagenmakers (2014).

Connectivity	Difference	t	d	p	p (FDR)	BF10	BF evidence
**sham planning vs tVNS planning**							
ATL <> PTL	.03 ± .04	.61	.09	.543	.63	.18	moderate H0
ATL <> IC	-.03 ± .05	-.48	.07	.63	.63	.17	moderate H0
ATL <> IFC	-.03 ± .04	-.67	.09	.508	.63	.19	moderate H0
PTL <> IC	-.09 ± .04	-2.14	.3	.037	.222	1.25	anecdotal H1
PTL <> IFC	-.03 ± .04	-.73	.1	.471	.63	.2	moderate H0
IC <> IFC	.04 ± .06	.71	.1	.481	.63	.2	moderate H0
**sham perception vs tVNS perception**							
ATL <> PTL	.04 ± .04	.99	.14	.327	.392	.24	moderate H0
ATL <> IC	.09 ± .06	1.47	.21	.148	.392	.42	anecdotal H0

ATL <> IFC	-.03 ± .05	-.67	.1	.504	.504	.19	moderate H0
PTL <> IC	-.04 ± .04	-1.07	.15	.29	.392	.26	moderate H0
PTL <> IFC	-.07 ± .04	-1.56	.22	.125	.392	.48	anecdotal H0
IC <> IFC	-.05 ± .05	-1.03	.15	.309	.392	.25	moderate H0

Supplemental Table S3

Contrasts of bilateral differences of non-linear connectivities, related to Results

Comparison of bilateral differences of linear connectivities values between tVNS and sham condition for action planning and perception phase, using frequentist and Bayesian t-test. Mean difference and SEM are given for each contrast beside t-value (t), Cohen's d (d), p-value (p) and FDR corrected p-value (p (FDR)). The Bayes factor strength of evidence label is given as evidence towards H1 if BF10 > 1 and otherwise as evidence towards H0 based on Lee and Wagenmakers (2014).

Connectivity	Difference	t	d	p	p (FDR)	BF10	BF evidence
**sham planning vs tVNS planning**							
ATL <> PTL	.04 ± .05	.71	.1	.481	.488	.2	moderate H0
ATL <> IC	.06 ± .07	.84	.12	.404	.488	.22	moderate H0
ATL <> IFC	.08 ± .06	1.38	.2	.174	.347	.37	anecdotal H0
PTL <> IC	.04 ± .05	.7	.1	.488	.488	.19	moderate H0
PTL <> IFC	.18 ± .05	3.59	.51	.001	.005	36.76	very strong H1
IC <> IFC	.09 ± .06	1.40	.2	.169	.347	.38	anecdotal H0
**sham perception vs tVNS perception**							
ATL <> PTL	-.12 ± .07	-1.7	.24	.096	.144	.58	anecdotal H0
ATL <> IC	.09 ± .07	1.28	.18	.207	.249	.33	moderate H0
ATL <> IFC	.11 ± .06	1.72	.24	.092	.144	.6	anecdotal H0
PTL <> IC	.14 ± .05	2.75	.39	.008	.025	4.43	moderate H1
PTL <> IFC	.20 ± .06	3.43	.49	.001	.007	23.76	strong H1
IC <> IFC	.05 ± .06	.83	.12	.411	.411	.21	moderate H0
